# Axial Length Growth Reference Curves and LMS Parameters for Japanese Children and Adolescents Aged 4–20 Years: The TMM BirThree Cohort Study

**DOI:** 10.1007/s44402-026-00133-0

**Published:** 2026-06-27

**Authors:** Masayuki Kobayashi, Mami Ishikuro, Taku Obara, Naoko Takada, Shunsuke Fujioka, Saiko Matsumura, Genki Shinoda, Aoi Noda, Keiko Murakami, Masatsugu Orui, Sayaka Yoshida, Akiko Hanyuda, Ryo Kawasaki, Atsushi Hozawa, Toru Nakazawa, Nobuo Fuse, Shinichi Kuriyama

**Affiliations:** 1 Graduate School of Medicine, Tohoku University, Sendai, Japan; 2 Chiba Kaihin Municipal Hospital, Chiba, Japan; 3 Tohoku Medical Megabank Organisation, Tohoku University, Sendai, Japan; 4 Tohoku University Hospital, Sendai, Japan; 5 School of Medicine (Omori), Toho University, Tokyo, Japan; 6 Graduate School of Medicine, The University of Tokyo, Tokyo, Japan; 7 International Research Institute of Disaster Science, Tohoku University, Sendai, Japan; 8 Keio University School of Medicine, Tokyo, Japan; 9 Graduate School of Medicine, University of Osaka, Osaka, Japan

**Keywords:** Axial length, Children, Growth curve, Japan, LMS method

## Abstract

**Purpose:**

Although axial length (AL) growth reference curves exist for several populations, there are no AL growth reference curves or established methods for AL-for-age *z*-score calculation for Japanese children and adolescents. This study aimed to develop AL growth reference curves and lambda-mu-sigma (LMS) parameters for Japanese children and adolescents aged 4–20 years, enabling a standardised evaluation of ocular development in both clinical and research settings.

**Methods:**

A cross-sectional analysis was conducted of the AL data from 14,482 children (7457 boys and 7025 girls) enroled in the Tohoku Medical Megabank Project Birth and Three-Generation Cohort Study. Optical biometry was performed using 10 consecutive measurements per eye and the mean values used to reduce measurement variability. Participants with a history of ophthalmic diseases that could affect AL were excluded. The LMS method with a Box-Cox-Cole-Green distribution was used to construct smoothed references and estimate the LMS parameters using the gamlss package in R.

**Results:**

Age- and sex-specific LMS values for the AL *z*-score calculation were provided at 0.1-year intervals to allow detailed age-specific assessment. Percentile and standard deviation curves were generated for each sex across the ages of 4–20 years. AL growth patterns in Japanese children were broadly comparable to those reported in other East Asian populations.

**Conclusion:**

The AL growth reference curves and LMS parameters established in this study offer a clinically relevant framework for evaluating ocular growth and monitoring myopia-related axial elongation in the Japanese paediatric population. These references contribute to early identification and intervention efforts in childhood myopia management and international comparisons.

Key Points

- Myopia is rapidly increasing worldwide, including Japan, amongst school-aged children.
- This study established axial length growth reference curves and lambda-mu-sigma parameters for Japanese children and adolescents.
- This study offers a clinically relevant framework for evaluating ocular growth and monitoring myopia-related axial elongation in Japanese children and adolescents.

## Introduction

The prevalence of myopia is increasing rapidly worldwide, particularly in developed countries, and its prevalence is significantly higher in East Asia, including Japan, than in Europe [1]. This trend is especially concerning in school-aged children, for whom early intervention can help slow myopic progression and reduce long-term complications, such as retinal detachment and glaucoma [2]. Given its increasing prevalence, myopia has become an important public health concern requiring timely and effective strategies [1, 2].

A major structural driver of myopia development and progression is the elongation of axial length (AL) [1], which is a growth-dependent biometric parameter in ophthalmology, analogous to height and weight in paediatrics. As AL changes continuously with growth, age-independent cutoff values can obscure gradual developmental changes and may lead to the misclassification of physiological versus pathological elongation. Therefore, employing age-specific reference values and thresholds is essential for evaluating AL accurately in children and adolescents, ensuring that measurements reflect physiological ocular growth and enable the precise assessment of myopia-related axial elongation.

Growth charts are widely used in clinical practice to assess anthropometric development and in public health to monitor generational trends by comparing data across different periods, and AL-for-age *z*-scores can be regarded as analogous tools for ocular growth assessment. As growth patterns differ according to sex and ethnicity, growth charts should be developed and applied considering these categorical factors to capture population-specific variations in growth patterns. Growth charts, on which individual growth curves can be plotted, are valuable for determining whether a child’s growth falls within normal limits and for visualising individual trajectories relative to population data. This enables clinicians to detect early signs of the disease and implement targeted preventive or management interventions when deviations from expected growth are identified. AL growth charts have already been developed for several ethnicities and regions, including Europe, China and Ireland, and these charts exhibit ethnic, regional and sex-specific differences in growth patterns [3–7]. However, to date, no AL growth charts have been reported for Japanese children and adolescents, and only a limited number of studies have provided methodologies for calculating AL-for-age *z*-scores.

Growth charts can be classified into two categories: standard and reference. The growth standard represents the growth pattern in a healthy population under optimal conditions, whereas the growth reference describes the actual distribution of anthropometric measurements in the general population, including individuals with various health and environmental conditions [8]. The Lambda-Mu-Sigma (LMS) method is a well-established statistical approach for constructing growth charts and corresponding *z*-scores [8]. Using the Box-Cox-Cole-Green (BCCG) distribution, this method models age-specific distributions of measurements, particularly when the data are skewed. The three parameters *L* (lambda), *M* (mu) and *S* (sigma) represent the Box-Cox power (related to skewness), median and coefficient of variation, respectively. By modelling *L*, *M* and *S* as smooth functions of age, this approach enables the calculation of age-specific *z*-scores, allowing a precise assessment of growth relative to peers of the same age. The *z*-scores for age are calculated using the following formula:

$$Z=\frac{{\left(X/M\right)}^{L}-1}{{LS}},$$

where *X* is the observed value and *L*, *M* and *S* are age-specific LMS parameters.

This study aimed to establish age‑specific AL reference data for Japanese children and adolescents aged 4–20 years. The goal was to develop LMS references for AL-for-age *z*-scores, along with percentile and standard deviation (SD) references, to facilitate the rapid and clinically meaningful assessment of ocular growth and myopia-related axial elongation in routine practice.

## Methods

Data were used from the Tohoku Medical Megabank Project Birth and Three-Generation Cohort (TMM BirThree Cohort) Study, as described elsewhere [9]. The TMM BirThree Cohort Study is a prospective cohort study conducted in Miyagi Prefecture and part of Iwate Prefecture, Japan, to assess the complex interactions of genomic and environmental factors in personal and preventive medicine. Between July 2013 and March 2017, 23,406 pregnant women were recruited from obstetric clinics or hospitals when their deliveries were scheduled. Subsequently, the newborns’ siblings, fathers, grandparents and other family members were also enroled [9]. Participants aged 4–19 years underwent physiological assessments at the research facilities, including AL measurements, using an OA-1000 or OA-2000 Optical Biometer (Tomey Corporation, tomey.de/products/oa-2000). Licensed nurses or medical technologists conducted 10 measurements for each eye and used the mean values to reduce measurement variability.

The AL data of children (initially recruited as foetuses) and their siblings were analysed, originally comprising 23,149 and 9459 individuals, respectively. Participants were excluded if they withdrew consent, had missing questionnaires or medical records, had ophthalmological conditions that could affect the AL measurements or lacked data on sex or height, as height is a fundamental anthropometric variable in the cohort and its absence could compromise the reliability of related analyses. Ophthalmological conditions were identified from medical records and questionnaires, and included congenital glaucoma, cataract, corneal opacity and other ocular diseases. Questionnaire information was used only to determine these exclusion criteria. After applying these criteria, AL data were available for 14,482 children and their siblings. The final dataset included AL measurements of 14,914 eyes of 7457 boys and 14,050 eyes of 7025 girls. The present analyses used cross-sectional data from the TMM BirThree Cohort Study, including all available AL measurements for each child. The inclusion criteria and dataset were the same as those used in our previous report [10]. Age-specific distributions are shown in Supplementary Table 1. The sample was skewed towards younger children, with particularly large numbers at 4–8 years of age, and progressively smaller numbers in the older age groups, especially from around 14 to 19 years of age. For each sex, there were generally more than 100 participants at each age between 4 and 11 years, whereas the numbers at ages 14–15 and 17–19 years were <50 participants at each age. Mean AL and SD values by age and sex were reported previously [10].

The LMS values for AL growth reference curves in boys and girls aged 4–20 years were estimated to create curves applicable to both eyes. Calculations were performed using the gamlss package (version 5.4.20) in R (version 4.3.2, r-project.org), which implements generalised additive models for location, scale and shape (GAMLSS). The model employed the BCCG distribution without logarithmic age transformation and smoothing parameters were selected by minimising the generalised Akaike information criterion GAIC(k) with a penalty *k* = 4 per effective degree of freedom to favour smoother centile curves and reduce overfitting.

## Results

**Fig. 1:**
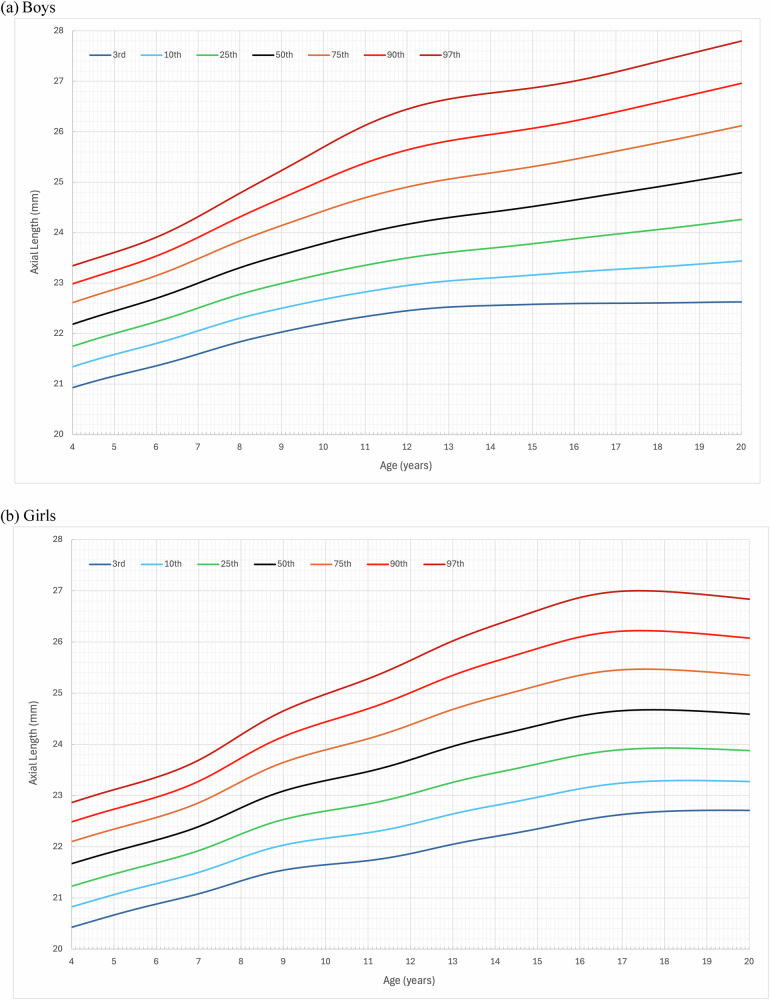
Axial length growth reference curves for Japanese children and adolescents aged 4–20 years. Boys (**a**) and girls (**b**) are shown with percentile curves (3rd, 10th, 25th, 50th, 75th, 90th and 97th).

The LMS values for the AL-for-age *z*-score calculation are shown in Table 1 at 1-year intervals and in Supplementary Table 2 at 0.1-year intervals. Percentile and SD values for the AL growth reference curves are presented in Table 2 and the corresponding percentile curves are shown in Fig. 1. Throughout childhood, boys consistently exhibited longer AL than girls, with the percentile curves for boys lying above those for girls across the entire age range and showing similar age‑related growth patterns. From late adolescence onwards, AL appeared to plateau in girls across all percentiles, whereas boys showed continued axial elongation into late adolescence. The AL distribution in this cohort has been described in detail in our previous report [10]. In brief, <10% of children under 13 years of age had ALs longer than 26.0 mm, whereas from early adolescence onward, a substantial proportion of eyes exceeded 24.5 mm [10].

**Table 1:** Lambda (*L*), Mu (*M*), Sigma (*S*) values of axial length growth reference curves for Japanese children and adolescents aged 4–20 years, boys and girls.

[Boys]			
Age (years)	*L*	*M* (mm)	*S*
4	2.568	22.187	0.0288
5	2.932	22.446	0.0288
6	2.858	22.700	0.0297
7	2.114	22.999	0.0313
8	0.927	23.305	0.0336
9	−0.359	23.557	0.0360
10	−1.511	23.786	0.0387
11	−2.250	23.992	0.0412
12	−2.520	24.164	0.0429
13	−2.355	24.298	0.0441
14	−1.870	24.405	0.0451
15	−1.229	24.517	0.0460
16	−0.582	24.644	0.0473
17	−0.101	24.777	0.0491
18	0.202	24.907	0.0510
19	0.482	25.043	0.0528
20	0.791	25.185	0.0546

[Girls]			
Age (years)	*L*	*M* (mm)	*S*
4	1.686	21.668	0.0299
5	1.511	21.908	0.0297
6	1.335	22.130	0.0297
7	1.160	22.393	0.0310
8	0.985	22.760	0.0334
9	0.810	23.088	0.0358
10	0.634	23.293	0.0381
11	0.459	23.469	0.0402
12	0.284	23.699	0.0423
13	0.109	23.961	0.0441
14	−0.066	24.172	0.0454
15	−0.242	24.367	0.0464
16	−0.417	24.553	0.0470
17	−0.592	24.658	0.0468
18	−0.767	24.674	0.0460
19	−0.943	24.642	0.0451
20	−1.118	24.591	0.0443

*L* and *S* are dimensionless parameters.

**Table 2:** Percentiles and standard deviation (SD) values of axial length (mm) growth reference curves for Japanese children and adolescents aged 4–20 years, boys and girls.

[Boys/Percentile]							
Age (years)	3	10	25	50	75	90	97
4	20.930	21.343	21.749	22.187	22.611	22.983	23.341
5	21.160	21.586	22.002	22.446	22.875	23.248	23.605
6	21.362	21.805	22.237	22.700	23.145	23.533	23.905
7	21.595	22.053	22.507	22.999	23.480	23.903	24.313
8	21.837	22.304	22.778	23.305	23.833	24.309	24.780
9	22.031	22.502	22.994	23.557	24.139	24.680	25.231
10	22.199	22.676	23.185	23.786	24.428	25.045	25.693
11	22.338	22.825	23.354	23.992	24.691	25.379	26.124
12	22.453	22.952	23.499	24.164	24.901	25.637	26.444
13	22.526	23.043	23.610	24.298	25.059	25.817	26.646
14	22.558	23.102	23.694	24.405	25.181	25.944	26.765
15	22.580	23.159	23.781	24.517	25.305	26.065	26.867
16	22.597	23.219	23.877	24.644	25.451	26.213	27.002
17	22.603	23.272	23.972	24.777	25.612	26.390	27.183
18	22.608	23.321	24.062	24.907	25.775	26.578	27.389
19	22.619	23.377	24.159	25.043	25.943	26.767	27.594
20	22.629	23.437	24.262	25.185	26.115	26.959	27.797

[Girls/Percentile]							
Age (years)	3	10	25	50	75	90	97
4	20.426	20.828	21.229	21.668	22.102	22.487	22.863
5	20.666	21.066	21.467	21.908	22.345	22.734	23.115
6	20.880	21.281	21.685	22.130	22.573	22.969	23.357
7	21.081	21.500	21.924	22.393	22.860	23.280	23.693
8	21.333	21.787	22.248	22.760	23.272	23.733	24.188
9	21.543	22.033	22.531	23.088	23.647	24.152	24.653
10	21.648	22.167	22.698	23.293	23.894	24.440	24.982
11	21.728	22.275	22.836	23.469	24.111	24.696	25.282
12	21.865	22.438	23.029	23.699	24.382	25.010	25.640
13	22.047	22.642	23.258	23.961	24.682	25.348	26.021
14	22.200	22.809	23.444	24.172	24.924	25.623	26.332
15	22.349	22.968	23.618	24.367	25.145	25.873	26.616
16	22.510	23.133	23.791	24.553	25.349	26.098	26.869
17	22.629	23.246	23.898	24.658	25.457	26.212	26.993
18	22.690	23.290	23.928	24.674	25.462	26.210	26.988
19	22.710	23.293	23.914	24.642	25.416	26.153	26.922
20	22.709	23.275	23.879	24.591	25.350	26.076	26.837

[Boys/SD]					
Age (years)	−2	−1	0	1	2
4	20.846	21.533	22.187	22.812	23.411
5	21.073	21.781	22.446	23.076	23.674
6	21.271	22.007	22.700	23.355	23.978
7	21.503	22.265	22.999	23.708	24.394
8	21.745	22.524	23.305	24.088	24.873
9	21.939	22.728	23.557	24.427	25.342
10	22.107	22.908	23.786	24.754	25.827
11	22.245	23.065	23.992	25.052	26.281
12	22.359	23.200	24.164	25.286	26.616
13	22.428	23.300	24.298	25.456	26.822
14	22.454	23.371	24.405	25.582	26.938
15	22.468	23.443	24.517	25.707	27.034
16	22.476	23.521	24.644	25.855	27.163
17	22.472	23.594	24.777	26.026	27.344
18	22.468	23.662	24.907	26.203	27.552
19	22.470	23.738	25.043	26.383	27.760
20	22.469	23.819	25.185	26.567	27.964

[Girls/SD]					
Age (years)	−2	−1	0	1	2
4	20.346	21.014	21.668	22.309	22.938
5	20.586	21.252	21.908	22.554	23.191
6	20.800	21.469	22.130	22.785	23.434
7	20.997	21.697	22.393	23.085	23.775
8	21.242	22.001	22.760	23.519	24.279
9	21.445	22.264	23.088	23.918	24.753
10	21.545	22.413	23.293	24.186	25.091
11	21.621	22.534	23.469	24.424	25.399
12	21.752	22.710	23.699	24.717	25.767
13	21.931	22.926	23.961	25.038	26.157
14	22.081	23.101	24.172	25.296	26.476
15	22.228	23.267	24.367	25.532	26.767
16	22.388	23.435	24.553	25.747	27.026
17	22.510	23.545	24.658	25.858	27.153
18	22.573	23.583	24.674	25.859	27.147
19	22.597	23.577	24.642	25.806	27.081
20	22.600	23.551	24.591	25.734	26.994

Percentile columns correspond to the 3rd, 10th, 25th, 50th, 75th, 90th and 97th percentiles, and SD columns correspond to *z*-scores of −2, −1, 0, 1 and 2.

## Discussion

This present work established AL growth reference curves for Japanese children and adolescents aged 4–20 years using the LMS method. The findings provide age- and sex-specific LMS parameters for calculating AL-for-age *z*-scores, with percentile curves provided as visual references. Detailed quantitative comparisons of age- and sex-related patterns of AL in this population have been presented in our earlier report [10].

These AL growth reference curves describe the distribution of AL in the general paediatric population and therefore should be interpreted as population references rather than growth standards based solely on emmetropic eyes. Recently, AL growth charts have been developed. Tideman et al. developed European AL growth reference curves from 4 years to adulthood using data from England and the Netherlands [3]. Although this is an important and large-scale study of European AL growth charts, they were age-adjusted using linear regression and, therefore, may deviate from the actual distribution. Additionally, their curves were expressed as normative values; however, these are likely to be references rather than standards based on the ideal conditions for AL growth [3]. McCullough et al. used a latent growth mixture model to estimate the Irish AL growth chart but did not directly provide methods for calculating AL-for-age *z*-scores [4]. Sanz Diez et al. constructed AL references for Chinese schoolchildren aged 6–15 years and supported the need for ethnicity-specific growth charts [5]. Although the smoothing method was not described in their 2019 study [5], the LMS method was used in their growth curve published in 2022 [6]. Their research may have been the first application of the LMS method to construct AL growth charts [6]. He et al. developed AL and AL/corneal curvature growth charts for Chinese children and adolescents aged 4–18 years [7]. Selecting growth charts for AL measurements requires careful consideration of factors, such as ethnicity and regionality, similar to the approach used for weight and height. The present study provides population-specific references for Japanese children and adolescents.

In myopia research, ethnic and environmental factors should both be considered when interpreting population differences in ocular growth [10]. Given the increasing prevalence of myopia, population-specific AL references may be useful for monitoring ocular growth over time. These reference curves may provide a useful basis for future AL-based myopia research, particularly by enabling age- and sex-specific assessment of ocular growth using AL-for-age *z*-scores. They may also allow more comprehensive analyses of genetic and environmental factors associated with myopia and childhood eye health within the TMM BirThree Cohort Study.

This study had some limitations. First, AL measurements were not obtained through longitudinal follow-up of the participants; rather, reference curves were based on cross-sectional data. Thus, these growth curves represent differences in AL across age groups in the current generation rather than individual longitudinal changes. Accordingly, their primary use is to assess an individual child’s AL relative to age- and sex-specific population distributions, rather than to predict individual growth trajectories or treatment effects. Given the increasing prevalence of myopia, age-specific AL distributions may change over time, and therefore, the applicability of these references to later cohorts should be interpreted with caution. In addition, the age distribution was unbalanced, with relatively few participants in the oldest age groups, in particular in the 15–19-year age range. Although the LMS method uses smoothing across the full age range, percentile estimates at these ages may be less stable and should be interpreted with caution. These factors may be particularly relevant to the finding that AL in girls did not follow a monotonically increasing trend. Second, because this study focused on biometric data, monogenic or syndromic forms of myopia were not specifically screened for, and such rare conditions may not have been excluded completely. However, medical histories were collected, not only from medical records and questionnaires, but also from routine health check-up records in infancy and at school age; thus, children with ocular conditions requiring clear clinical intervention were likely to have been excluded. Finally, this study was conducted in specific regions (Miyagi and Iwate Prefectures), and the results may not be generalisable to all regions of Japan. However, the study area includes both rural communities and an urban centre, Sendai City, thereby providing a broad range of living environments. Nevertheless, future studies in other settings are required to confirm the generalisability of the findings.

## Conclusion

In this study, AL growth reference curves were established for Japanese children and adolescents aged 4–20 years using the LMS method. These references and LMS parameters enable age- and sex-specific assessment of AL using AL-for-age *z*-scores. Additionally, the reference curves provide a foundation for regional and international comparisons of AL growth and can facilitate broader epidemiological and clinical research.

## Supplementary information

SupTable1_age_dist_20260603.

SupTable2_LMS0.1_20260604.

## Data Availability

The data supporting the findings of this study are not publicly available because of privacy and ethical restrictions. The data may be made available by the corresponding author upon reasonable request and with the approval of the TMM Sample and Data Access Committee.
